# Verrier’s and Schiltsky’s Continuous Gum Methods

**Published:** 1886-02

**Authors:** A. Witzel


### VERRIER'S AND SCHILTSKY'S CONTINUOUS GUM METHODS.

BY DR. A. WITZEL.

READ AT THE THIRTEENTH ANNUAL MEETING OF THE AMERICAN DENTAL SOCIETY OF EUROPE, BERLIN, AUGUST, 1885.

Continuous gum-work, which I consider to be the most difficult but most artistic that can be done in mechanical dentistry, has unfortunately found its way into only a few dental laboratories. The necessity of having specially prepared teeth for this work has, for for many practitioners, been a great objection. In experimenting with various teeth and different preparations of gum body and enamel, I have been lead to the conclusion that great improvements may be made in this direction. Also the different ingeniously invented furnaces leave much to be desired. I have found the one muffle furnace, with the annealing stand of Mr. B. A. Verrier, to give in my hands the best results. Not having a very large selection of continuous gum teeth, I have been obliged to use the ordinary plate teeth prepared for metal plates. These I have successfully employed in the following manner:

After the teeth are fastened with wax on their labial side to the well fitted and roughened platinum plate, I insert the whole in equal parts of plaster and asbestos, so that only the labial side remains free. After removing the wax with boiling water, I replace it with Dr. Allen's gum body, and fire it in the usual way. After the second baking, I remove the investment, turn the rim with a burnisher, and fill up any defects behind the teeth with gum body. Then I generally put a fresh investment of plaster and asbestos on the palatine side.

To get the right temperature for baking the gum enamel has been another great difficulty; but by using Mr. Verrier's furnace with the right pressure of gas, working Mr. Fletcher's foot bellows at the same rate, watching the time required to melt the gum enamel after the melting point of a piece of pure gold which I put in the muffle with the piece to be fired has been reached, I always get equally good results in from ten to twelve minutes.

After the investment is removed, and when I wish to strengthen the continuous gum facing before vulcanizing, I solder a piece of 18 K. wire to the backs of the teeth. By the use of the much more fusible gum body and enamel made by Mr. O. Schiltsky, of Berlin, I have been able to make a few nice partial pieces with any kind of teeth. But the great amount of shrinkage, the color and glassy appearance of this composition, leave me to give the preference to Dr. Allen's preparation. The small muffles of Mr. O. Schiltsky's furnace allow only the manufacture of blocks, which is also a great disadvantage. However, if we compare the disadvantages of both compositions with their usefulness, I consider that no well mounted laboratory should be without the one or the other, and with sufficient practice I am sure that the difficulties of continuous gum working will soon be conquered.

